# MicroRNA Target Identification: Revisiting Accessibility and Seed Anchoring

**DOI:** 10.3390/genes14030664

**Published:** 2023-03-07

**Authors:** Nicolas Homberg, Mariana Galvão Ferrarini, Christine Gaspin, Marie-France Sagot

**Affiliations:** 1 Laboratoire de Biométrie et Biologie Evolutive, Université de Lyon, CNRS, UMR5558, 69622 Villeurbanne, France; 2 INRIA Lyon Centre, 69100 Villeurbanne, France; 3 UR0875 MIAT, INRAE, Université de Toulouse, 31326 Castanet-Tolosan, France

**Keywords:** RNA-RNA interactions, microRNA, accessibility, conservation intra-species, CLASH-data

## Abstract

By pairing to messenger RNAs (mRNAs for short), microRNAs (miRNAs) regulate gene expression in animals and plants. Accurately identifying which mRNAs interact with a given miRNA and the precise location of the interaction sites is crucial to reaching a more complete view of the regulatory network of an organism. Only a few experimental approaches, however, allow the identification of both within a single experiment. Computational predictions of miRNA–mRNA interactions thus remain generally the first step used, despite their drawback of a high rate of false-positive predictions. The major computational approaches available rely on a diversity of features, among which anchoring the miRNA seed and measuring mRNA accessibility are the key ones, with the first being universally used, while the use of the second remains controversial. Revisiting the importance of each is the aim of this paper, which uses Cross-Linking, Ligation, And Sequencing of Hybrids (CLASH) datasets to achieve this goal. Contrary to what might be expected, the results are more ambiguous regarding the use of the seed match as a feature, while accessibility appears to be a feature worth considering, indicating that, at least under some conditions, it may favour anchoring by miRNAs.

## 1. Introduction

MicroRNAs (miRNAs for short) represent one of the best-studied entities among a vaster class of non-coding RNAs called small non-coding RNAs (sncRNAs). They play essential roles in eukaryotes by regulating their target gene’s expression [1]. For any given miRNA, this role may go beyond the limits of a single cell. It was indeed shown that miRNAs are able to move inter-cellularly through body fluids such as blood, tears, and maternal milk [2,3,4]. More recently, it was even hypothesised that miRNAs from a eukaryotic species may target cross-kingdom organisms living in close contact [5,6].

In this paper, we concentrated our attention on the problem of intra-species miRNA target identification. Indeed, although this may look like a simpler problem that furthermore has been much studied already, many open questions remain, as well as some contention points related to the estimated number of false positives [7,8]. Moreover, there is a lack of unanimity on the criteria that should be used to infer the targets of miRNAs. The main objective of this paper is thus to explore in finer detail some of the open problems that continue to haunt the in silico approaches for miRNA target identification, knowing that these continue to be important, as experimental approaches, on the other hand, remain expensive in terms of both cost and time. Experimental datasets do, however, exist, notably for miRNAs that have been studied for a longer time.

Some studies try to detect regulation through a direct correlation of expression between miRNA and mRNA in diverse conditions [9]. These results, however, do not indicate the exact sites of interaction. This is why the two that will be used in our case correspond to the CLASH (for Cross-linking, Ligation, And Sequencing of Hybrids) data presented in the paper of Helwak et al. [10], in which the authors identified human miRNAs along with their mRNA interaction sites within a single experiment, as well as the CLASH mouse dataset presented in the paper of Moore et al. [11].

A high number of features have been explored in the literature when trying to computationally infer the potential targets of miRNAs, as high as, e.g., 26 in one of the best-known methods TargetScan [12]. Among such features, some of the main ones that have been considered include: (1) a match with what is called the seed sequence in the miRNA, which is usually defined as starting at nucleotide 2 from the 5’ end of the miRNA and is of a length between 6 and 8; (2) the stability of the duplex formed between the miRNA and the mRNA, which can be in the 3′UTR, the CDS, or the 5′UTR of the target mRNA; (3) the accessibility of such a site within the mRNA or of the close region around it; and (4) evolutionary conservation of the miRNA and mRNA sequences.

Among such features, we chose to focus exclusively on the seed and on the accessibility of the targeted mRNA region, starting with the accessibility feature. Among the currently better known, and most used methods for miRNA target prediction, it is indeed notable that some use such a feature, while others do not. We therefore decided to explore this issue.

As concerns the seed, we asked two main questions. The first was the same as for the accessibility, meaning we wished to compare the results obtained when such a feature is used or not. The second question was related to the fact that, in general, a given miRNA may interact with many mRNA locations in any given organism, most often in a similar way, meaning that the seed region on the miRNA is the same, and thus the sites with which it interacts should have some resemblance among them. This means that such locations could be seen as the approximate occurrences of a motif that should be the reverse complement of the seed. The point we wanted to investigate, then, was whether looking for motifs that appear conserved across different mRNA sequences in the same species, under some definition of conservation, might represent a feature to be considered when trying to infer miRNA–mRNA interaction sites.

As for both investigations, we used CLASH data, and we start by briefly presenting it in the next section.

## 2. Materials and Methods

### 2.1. Datasets

Among the published CLASH data, we chose to use those from two studies that provide information on the precise sites of interaction between the miRNAs and their mRNA targets. Experimental validation methods based on CLASH are the only ones that do not rely on in silico predictions to find the interacting miRNAs. Indeed, in such cases, the miRNAs are linked and sequenced along with their mRNA targets.

In the present study, the input for the in silico methods used is always a set of miRNA–mRNA couples where, for each couple, at least one miRNA interaction site has been identified by CLASH in the mRNA (sometimes more than one). We then decided to proceed in two ways.

In the first case, the complete mRNA sequences along with their interacting miRNAs were provided as input to the methods, and in the second one, only a window around the interaction site in the mRNA was considered.

We denoted by Seq${}_{C}$ the dataset with complete mRNA sequences and by Seq${}_{W}$ the dataset including the interaction site plus 200 nucleotides down- and upstream of said site. The first case may thus be considered as containing more noise, and the idea was to also see whether this influenced the behaviour of the different methods and the results obtained.

Notice that by giving as input the exact miRNA–mRNA couples identified by the CLASH experiments, **we never consider miRNA–mRNA couples where the miRNA is not known to interact**. This means that **there are never any true negatives** in the results obtained. Moreover, any false positives are related not to the couple itself, **but to the exact location of the interaction site on the mRNA**.

The miRNA–mRNA interactions provided by the CLASH experiments were supported by chimeric reads, which were composed of the merge of the sequences of an miRNA and of an mRNA. The amount of these reads is a good indicator of the strength of the interaction.

#### 2.1.1. Human CLASH Data

The information provided by the data from this first study is detailed in [10]. It describes the precise placement of 399 human miRNAs onto 7390 mRNA targets, representing a total of 18514 miRNA–mRNA interactions.

The lengths of the mRNA subsequences as given by the authors to be involved in the interaction sites vary from 18 to 119, with an average of 52.8 nucleotides.

To generate the dataset Seq${}_{C}$, we proceeded as follows. From the version of the human genome used in the CLASH paper, namely *GRCh37 release 60* downloaded from the *Ensembl* [13] database, we selected only the sequences of interest from the cDNA by matching the name of the mRNA from CLASH and then extracting the mRNA sequences that contain an interaction site situated exactly where CLASH placed it. If several candidates from the cDNA corresponded to one same mRNA in CLASH, we selected the one with the longest sequence. In the process, two mRNAs from CLASH were thus not considered due to an inconsistency between the sequence provided in the paper and the sequence we retrieved from the database.

Thus, we ended up with a dataset Seq${}_{C}$ of 18,497 interactions from 399 miRNAs targeting 7388 mRNAs.

The dataset Seq${}_{W}$, on the other hand, is slightly smaller as indeed, we eliminated from it those sequences in which, when extending the precise interaction sites as given by CLASH of 200 nucleotides down and upstream, the distance of the site from the 5${}^{\prime }$ or the 3${}^{\prime }$ end of the mRNA was smaller than 200. For this Seq${}_{W}$ dataset, we obtained 15,754 interactions.

The interaction sites were further classified by the authors into different groups, either based on the region of the mRNA where they are located or on the class of interaction to which they belong. In relation to the regions, it is important to notice that although most often, only the 3′UTR regions of the mRNAs are considered by the in silico methods for inferring the miRNA targets, the CLASH data allowed us to report approximately 5% of the interaction sites within the 5U′TR regions and, surprisingly, 60% within the CDS regions, as compared to 35% within the 3′UTR.

**Figure 1:**
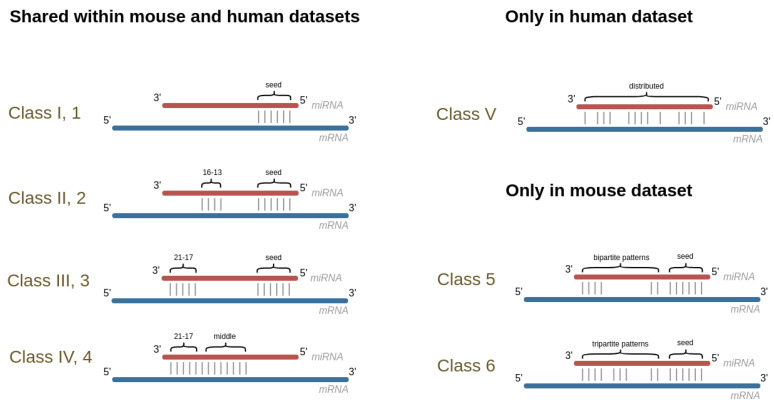
Representation of the classes as defined in the human [10] (I, II, III, IV and V) and in the mouse [11] (1, 2, 3, 4, 5, and 6) studies.

In the same way, considering the miRNAs now, the interaction sites are divided into five classes [10], three of which, namely Classes I, II and III, involve a seed at the 5${}^{\prime }$ end of the miRNA. This involvement is either strict (Class I) or concerns the seed plus a region of, respectively, 4 or 5 bases at a shorter (Class II) or slightly longer (Class III) distance from the seed. Regarding the other two classes, as indicated in the CLASH paper [10], the binding is either limited to a region located in the middle or the 3${}^{\prime }$ end of the miRNA (Class IV), or it is distributed and less stable (Class V). The proportion of targets in each class varies between approximately 17.8% for the smallest class (Class II) and 25% for the biggest one (Class III); see Figure 1.

We wish to call attention to the fact that while the numbers obtained for the classes add up, as expected, to the number of miRNA–mRNA interaction sites considered, this is not necessarily the case when we consider the regions instead of the classes. The reason is that some interaction sites may be located between two regions, namely 5${}^{\prime }$UTR and CDS or CDS and 3${}^{\prime }$UTR, or may be found in non-coding RNAs that arise from pseudogenes as indicated by the authors. When there exists a difference, it is a small one.

The number of interaction sites for each of the two datasets above, in total and distributed in regions and classes, is given in Table 1.

#### 2.1.2. Mouse CLASH Data

The data from the second study were produced by Moore et al. [11]. The study made available 130,119 *Mus musculus* interactions involving 614 miRNAs and their 11,748 target sequences, for which only the chromosome and positions on them were given.

The lengths of the mRNA subsequences involved in the interaction sites, as indicated by the authors, vary from 23 to 161, with an average of 45.6 nucleotides.

We first processed the target sequences in order to assign them to the 5${}^{\prime }$UTR, CDS, and 3${}^{\prime }$UTR regions. We retrieved the matching transcript names with annotation information that enabled us to associate such names with the positions on the chromosomes (from the mouse genome NCBIM37, Ensembl release 54 [13]). We retrieved for each transcript name the corresponding cDNA sequences (from Biomart version 54 [13]) and checked that the interaction sequence retrieved from the full chromosome could be identically aligned on the transcript sequence. When several mRNAs matched with their names and sequences, we chose the longest one. The resulting dataset includes 13,071 interactions between 378 miRNAs and 2462 mRNAs. The numbers of sequences for Seq${}_{W}$ and Seq${}_{C}$, in total and by region and class, are given in Table 2.

Notice that by focusing on the mRNA sequences, as in the human study, we eliminated a high number of interactions, such as miRNAs with introns and miRNAs with intergenic regions.

Six different classes are distinguished by the authors in this dataset. Classes 1, 2, 3, and 4 correspond to, respectively, Classes I, II, III, and IV as determined in the human study [10]. The other two classes, which involve the seed and a bi- or tripartite auxiliary pairing pattern, were defined as new ones, namely Class 5 and Class 6 (see Figure 1, [11]).

### 2.2. Revisiting Accessibility through CLASH Data

There are many methods for inferring miRNA–mRNA interactions. However, as we wanted to conduct our study using experimentally validated data, namely the CLASH human and mouse data presented in the previous section, this implied that, for any given method, we could run it ourselves on such data, which meant that the method should be publicly available. Furthermore, since our objective was to focus exclusively on two main features, namely accessibility and the seed, we decided to choose among such methods those that do not use many more features or ones that would introduce noise. Three main methods, which are also among the better-known ones, were thus identified and chosen. These are miRanda [14], Pita [15], and IntaRNA [16,17]. Notice that the last one, IntaRNA, is not specific to miRNA–mRNA interactions. It was indeed originally developed to identify sncRNA–mRNA interactions in bacteria. A fourth method, also very well known, could have been considered, as it published in its original paper a study based on the same CLASH data as we are using here. This is TargetScan [12]. The method, however, uses many other features, namely 26, including inter-species evolutionary conservation. We therefore put it aside to allow us to exclusively focus on the importance of the seed match and accessibility within our results.

Before briefly presenting the main characteristics of each of the methods selected, it is important to remember that we have one main motivation for this study, which is the issue of accessibility: is this an interesting feature or not? Two of the methods chosen use this feature, namely Pita, whose development was indeed strongly motivated by its importance for the authors (as reflected in its name, which stands for Probability of Interaction by Target Accessibility), and IntaRNA. The third method, miRanda, is thus the only one that does not explicitly compute the accessibility of the mRNA.

Moreover, we would like to observe that all methods, including the three that we chose to use, often include more general tools as modules within their algorithm; these tools are publicly available. Many belong to the ViennaRNA Package [18] and enable addressing some basic problems. These are related to the main features that are considered when trying to infer miRNA–mRNA interactions.

These main features concern:

- The seed match. We recall that this is the problem of finding the best binding site between two RNAs. It is addressed by adopting a sequence alignment method, albeit with some adaptation, the main of which is that sequence complementarity and not sequence identity is considered. We recall that in the case of miRNAs, the seed starts at nucleotide 2 from the 5${}^{\prime }$ end of the miRNA and is of a length between 6 and 8.
- The minimum free energy of the interaction, or what was called the stability of the duplex formed between the miRNA and the mRNA in the introduction. This corresponds to the secondary structure between miRNA and target mRNA that has the lowest value of free energy required to unfold it. Some methods further work within a nearest-neighbour free energy model, where dinucleotides instead of single nucleotides are considered [19]. The way free energy is computed may differ importantly between different methods [20,21].
- The accessibility of the interaction site. This is related to the free energy that would be required to open the secondary structure of the mRNA [15,22].

We then present below the main characteristics of the three methods that were used in our study:

- Pita: Among the methods for predicting the targets of a miRNA, Pita was a pioneer in considering accessibility when attempting to carry out such predictions. With that aim in sight, after identifying miRNA interaction sites using complementary sequences to the seed, it computes an energy score ΔΔG, which is equal to the difference between ΔGduplex and ΔGopen, the first being the energy for binding both RNAs together, which is computed with a modified version of RNAduplex, while the second is the energy required to open a region and is computed using RNAfold [23] and a sliding window of 70 additional nucleotides before and after the predicted target region.
- IntaRNA: Similar to Pita, IntaRNA predicts RNA–RNA interactions using the accessibility of the target sites. IntaRNA actually combines two energy scores, the first one representing the target accessibility computed with RNAplfold [23], and the second one corresponding to the energy of the RNA–RNA hybridisation computed as in RNAhybrid [20]. Again, as with Pita, IntaRNA does look for complementary sequences to the seed; however, contrarily to Pita, it does this in a third step only.
- miRanda: miRanda is the only method among the three chosen that does not consider the accessibility of the target region. The method proceeds by first searching for complementary sequences with an emphasis on binding the 5’ region of the miRNA (region of the seed) and then by computing the energy of the pairing using RNAduplex.

In order to revisit accessibility and seed match, we first evaluated whether or not the methods considering these features were better predictors than those not including them. In a second step, we focused on IntaRNA, which was used alone to allow us to consider or not each one of these features while computing predictions of miRNA–mRNA interactions.

### 2.3. Performance Evaluation

For each method, the performances were evaluated in terms of precision, recall and F-score metrics, considering the different numbers of chimeric reads at the interaction sites. On the other hand, we wanted to be very precise and thus decided to initially consider—in both cases of datasets—a position as a **strong true positive**, *TP*${}_{strong}$ for short, when the interaction site predicted by the method is **fully included within the site given by CLASH**. It may also happen that the predicted site overlaps the site given by CLASH without being fully included in it. This may be seen as a weaker type of true positive, and we therefore chose to also consider it separately. We named it a **weak true positive**, *TP*${}_{weak}$ for short.

We then called false positives, FP for short, all predictions made that are not *TP*${}_{strong}$. Note that FP is the same for both *TP*${}_{weak}$ and *TP*${}_{strong}$.

In the same way, there are different types of false negatives that may be considered for each miRNA–mRNA couple given as input. One will be a strong false negative in the sense that no interaction site whatsoever is detected. We denoted it by No-Prediction for short. We also have weaker types of false negatives when a prediction is made that does not fully fall within the interaction site as given by CLASH; it either overlaps such a site or is outside it (however by force corresponds to the right couple). These weaker false negatives plus the strong ones add up to what we named **all false negatives**, *FN*${}_{all}$ for short.

Given these distinctions between strong and weak true positives, we computed two types of precision (1), recall (2) and F-score (3).

(1) $$\mathrm{Precision-strong}=\frac{TP_{strong}}{TP_{strong}+FP}\;\mathrm{Precision-overlap}=\frac{TP_{strong}+TP_{weak}}{TP_{strong}+FP}$$

(2) $$\mathrm{Recall-strong}=\frac{TP_{strong}}{TP_{strong}+FN_{all}}\;\mathrm{Recall-overlap}=\frac{TP_{strong}+TP_{weak}}{TP_{strong}+FN_{all}}$$

(3) $$\begin{array}{cc} \mathrm{f-score-strong} & =2\frac{\mathrm{recall-strong}\times \mathrm{precision-strong}}{\mathrm{recall-strong}+\mathrm{precision-strong}}\\ \mathrm{f-score-overlap} & =2\frac{\mathrm{recall-overlap}\times \mathrm{precision-overlap}}{\mathrm{recall-overlap}+\mathrm{precision-overlap}} \end{array}$$

### 2.4. mRNA Accessibility

We used two approaches to compute the mRNA accessibility. In the first one, we defined mRNA accessibility to be related to the number of times a nucleotide was unpaired in all the optimal structures it belongs to, with each structure being computed inside a window of 150 nt sliding along Seq${}_{W}$.

In order to compute such structures, we used RNAfold from the ViennaRNA package. The output of RNAfold gives the minimum free energy of the optimal secondary structure as well as the structure in bracket notation. Using this notation, we can then count the number of times a nucleotide is paired or not when we slide the window, moving it nucleotide per nucleotide until the end of the mRNA sequences given as input. The second approach adopts another well-used definition of accessibility, which is implemented in RNAplFold [23].

The accessibility values of both methods were normalized to be in the range of 0 to 100.

### 2.5. Exploring Intra-Species Conservation

We used Meme [24] to explore intra-species conservation. We considered separately, for each miRNA in both CLASH datasets, the set of mRNAs it targets. In the first experiment, for each miRNA, we gave as input to Meme the exact interaction sites on its mRNA targets. This dataset is denoted by Seq${}_{E_{i}}$. In the second experiment, for each miRNA, we gave as input to Meme the exact site plus 200 nucleotides down- and upstream of that site for all its mRNA targets. This dataset is denoted by Seq${}_{W_{i}}$.

In both cases, we selected only the motif with the lowest combined match p-value [25], which we considered as the best one found by Meme.

We used the parameters by default from the tool as available on the website (meme-suite.org; version accessed on 9 September 2022, see the commands used in Table S1), except for the site distribution and the width of the motifs. As concerns the site distribution, indeed, we changed from the default one which searches for zero or one occurrence per sequence (zoops) to the one that searches for one occurrence per sequence (oops). As concerns now the width of the motif, by default, Meme sets it between 6 and 50 nucleotides. We changed this to between 6 (minimum length of a seed) and 23 (maximum length of a miRNA). The positions on each sequence for every motif occurrence were recovered directly from the output of Meme.

The performance of the motif predictions was also evaluated using the precision, recall and F-score metrics for the miRNAs with, respectively, at least two and at least ten targets. In that case, we considered the occurrence of a given motif predicted by the method as a **strong true positive**, *TP*${}_{strong}$ for short, when it is **fully included within the site given by CLASH**. When the occurrence of a predicted motif overlapped the site given by CLASH without being fully included in it, this was considered a weaker type of true positive, and we therefore chose to also consider it separately. We named it a **weak true positive**, *TP*${}_{weak}$ for short. Considering the methods used for motif prediction and the constraint of having only one solution, the false positives, FP for short, and false negatives, FN for short, have the same value. Notice that, as a consequence, the recall and the precision metrics have also the same value.

## 3. Results

### 3.1. Prediction of Interactions from the CLASH Datasets Using Three Methods

**Figure 2:**
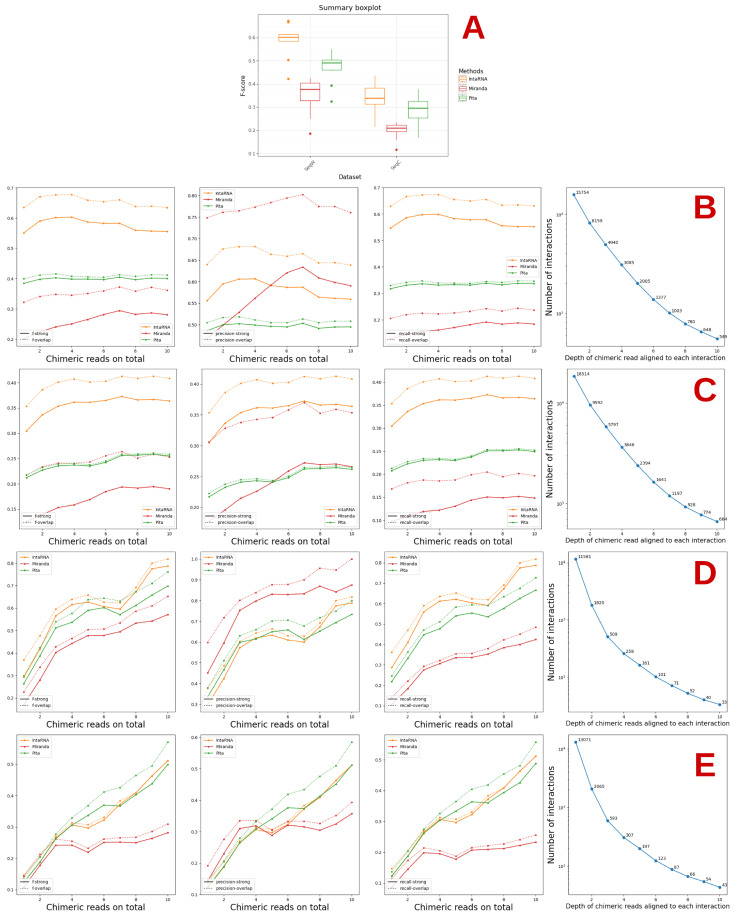
F-score, precision and recall for miRanda, Pita, and IntaRNA used with default parameters. (**A**) summarises the results of the combined human and mouse datasets. (**B**,**C**) and, resp., (**D**,**E**) show the results on the Seq${}_{W}$ and Seq${}_{C}$ human, resp. mouse, datasets.

The results obtained for the human and mouse CLASH datasets in each of the two cases of input considered, namely Seq${}_{C}$ or Seq${}_{W}$, are presented in Figure 2 for all regions and classes together and in the Supplementary Figures S1 and S3 for each region and each class.

We observe that the results obtained by any method on the miRNA–mRNA datasets are always worse in terms of precision or recall than those obtained when considering Seq${}_{W}$, and this occurs in a rather important way (see Figure 2). This is expected, as the amount of noise in the first case is, as mentioned, much higher. There indeed, we consider the whole sequence of the mRNAs for which at least one miRNA–mRNA interaction site has been validated by CLASH, while in the second, we consider only a small region around such interaction sites.

For the mouse dataset, the results improve as the number of reads increases.

Considering classes and regions, the poorest results overall are, in general, obtained for Classes IV and V for the human data as well as Classes 4 and 5 for the mouse data (see Supplementary Figures S1 and S3). This is expected as these classes are supposed to correspond to miRNAs that bind to the mRNA not through the seed, not even necessarily in a localised or strongly stable manner. For both datasets, the 3′UTR regions always exhibit the best results in terms of F-score.

Interestingly, concerning accessibility, miRanda, which does not consider this as one of its features has, with a few exceptions, the worst behaviour in relation to both Pita and IntaRNA, more substantially in terms of F-score and recall, whatever the type, than in terms of precision.

This appears to support the idea that accessibility is an important feature, although some caution should be taken as there are other differences among the methods analysed here, even when considering the same feature as the exact parameters used for such may differ. The order in which the features are considered, which is not always the same for all three methods, may also have an influence on the results. In any case, as concerns accessibility, this explains why we decided to refine our study, as presented in the next section.

### 3.2. Study of the Accessibility of the Interaction Site and Seed Match with *IntaRNA*

Before proceeding with a direct study of the accessibility of the interaction site, we decided to refine one of the studies made in the previous section, related to the method that in almost all cases produces the best results on the CLASH data, namely IntaRNA.

**Figure 3:**
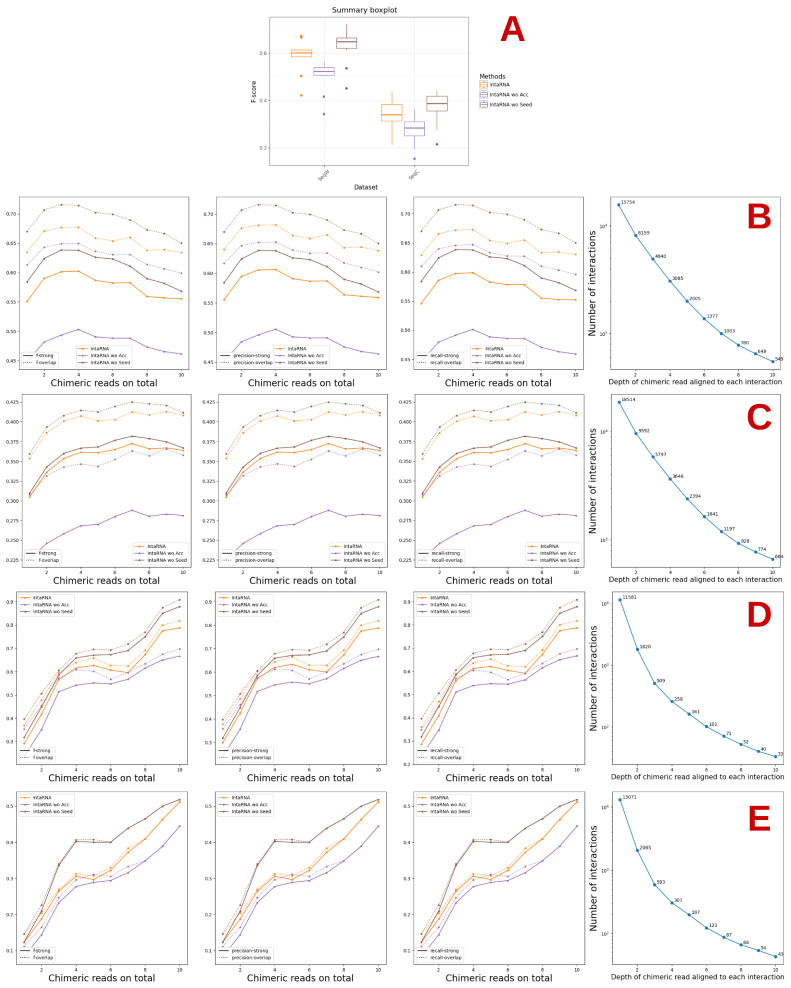
Performance of IntaRNA considering or not accessibility and seed match. The term wo in the Figure stands for WithOut. (**A**) summarises the results of the combined human and mouse datasets. (**B**,**C**) and, resp., (**D**,**E**) show the results on the Seq${}_{W}$ and Seq${}_{C}$ human, resp. mouse, datasets.

Such refinement concerned eliminating accessibility or seed matching. This is indeed possible with the new version of IntaRNA published in 2017 [17]. Results are presented in Figure 3. Here, over-start and over-end indicate the number of predictions made that were not fully within the interaction site but overlapped it, either at the start or at the end. The term wo in the Figure stands for without. To facilitate the comparison, we indicated again the results obtained by IntaRNA with its default parameters.

For both the human and the mouse datasets, the best results are obtained when considering the accessibility (Figure 3). There are rare exceptions in the case of the human data for Seq${}_{C}$ (Precision-overlap for the 5′UTR region) and five for Seq${}_{W}$ (Recall-strong for Total, 5${}^{\prime }$UTR, CDS, 3${}^{\prime }$UTR, and Class V; see the Supplementary Material, Figures S2 and S4). At least in relation to IntaRNA, this reinforces the idea that accessibility is an important feature and should be taken into account in the prediction of the interaction sites.

While the worst results are obtained when not considering the accessibility, not considering the seed gives either the same results as IntaRNA with default parameters or, in some cases, better results with some exceptions for Classes 1 and 6 on Seq${}_{W}$ of the mouse data, where the results without the seed became the worst when considering interactions supported by at least 6 and 8 chimeric reads, respectively (see the Supplementary Figures S2 and S4).

When the result is better without the seed, the difference is never very substantial. Such results are nevertheless somewhat unexpected and surprising. One possible explanation might be that IntaRNA considers the seed in the last step of its procedure. It would have been nice to be able to conduct the same test with Pita or miRanda, both of which start by checking for a match to the seed. There is, however, no way to fully eliminate such a feature, as is the case with IntaRNA. The only possibility, for instance, with Pita, would be to enter a very low value as a parameter for the seed width (in this case, 3), but this is a rather artificial way of proceeding and could thus generate other artefacts or bias the results in unexpected ways.

### 3.3. Refinement of the Accessibility Study

Although the results of the plots presented in Figure 3 in Section 3.2 reinforce the importance of considering the accessibility of an interaction site, we wished to further extend our exploration of this feature by using RNAfold and RNAplFold.

Given a window of length *l*, a given nucleotide in a given sequence may thus belong to a different structure at most *l* times, except for those at positions less than *l* or greater than L−l+1 of the mRNA, where *L* is the latter’s length. Considering all mRNAs of which there are *N*, a nucleotide may then be part of at most *l* times *N* structures. Following the literature, we fixed *l* at 150, which is the size of the window used in IntaRNA. As concerns *N*, it is equal to 15,754 for the human dataset and 11,581 for the mouse dataset, as indicated in Table 1 and Table 2.

The accessibility is then measured by a scoring procedure based on the computation of the average number of times each nucleotide is unpaired.

We also computed accessibility as implemented in RNAplFold [23]. We parameterized RNAplFold with a window size (*W*) of 150 nts, inside which the accessibility is computed, a maximum base pairing span (*L*) of 100 nts and a continuous accessible sequence (*u*) of 1 and 8.

**Figure 4:**
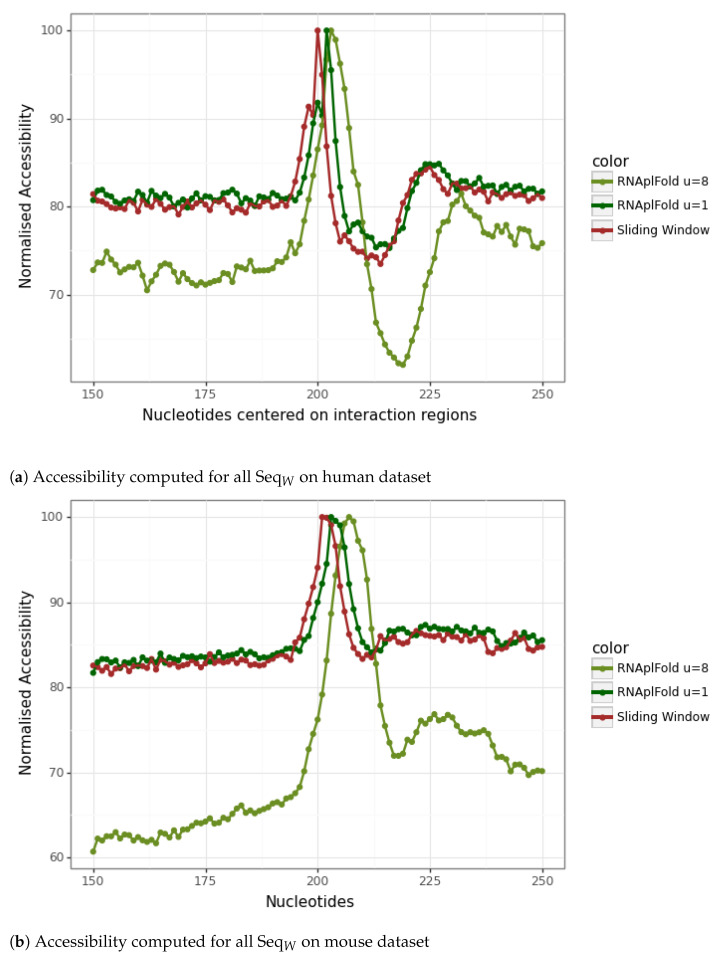
Accessibility computed for all Seq${}_{W}$ on mouse and human datasets. In red is the accessibility when considering only the optimal structure with a sliding window. In dark green and in olive green are the accessibility computed with RNAplFold with an accessible window *u* of 1 and 8.

Figure 4 shows the results obtained considering all 15,754 mRNAs. Each class is displayed separately in the Supplementary Figures S5 and S6. For the sake of clarity, the graphic is centered on the interaction site, and values are shown from position 150 to 250.

For both methods, we observe a rather distinct positive peak at the central position where the site is predicted to start by CLASH. This positive peak covers the start of the interaction and indicates a site potentially more accessible and thus more prone to beginning an interaction. However, if we look now at the same type of graphic but this time consider each mRNA sequence individually, it is difficult to observe any accessible regions as presented in the Supplementary Material, Figures S7 and S8. Notice that the accessibility approach using the sliding window (Red) only considers one optimal structure for each window, whereas the approach using RNAplFold takes into account all suboptimal structures (see Figure 4). Both approaches exhibit similar results.

Altogether, these results support the idea that accessibility is in general an important feature.

### 3.4. Exploring Intra-Species Conservation

Without having this specific aim as an objective, the possible conservation or over-representation of motifs **intra-species** has been examined already in the literature and notably in one of the papers presenting the CLASH data that we are using here [10]. Besides the fact that such studies have been quite high-level, there was never the idea that such conservation might be considered as an extra feature in the identification of interaction sites given that some miRNAs target many different mRNAs. One would then expect that, in such cases at least, taking into account such additional information could help improve the discovery of new interactions for an already known miRNA or even point to the existence of new miRNAs not yet identified. Clearly, as mentioned, such an idea will not concern all miRNAs, only those that have multiple targets.

The objective of this section is then to explore this idea in a more systematic way, still using the CLASH data. This exploration will involve first checking if one of the most, if not the most, universally used tool for inferring motifs, namely Meme and its suite [24,26], is able to associate the exact interaction sites of an miRNA with a statistically significant motif. As concerns the number of miRNAs, which were 399 in human CLASH data, only 316 were thus taken into account, as the remaining 83 ones (approximately 21% of the miRNAs) have only one predicted interaction site. When considering miRNAs that have at least 10 predicted interaction sites, the number goes down to 162 (approximately 41% of the total). Similarly, in the mouse dataset, only 299 of the 378 miRNAs have 2 or more targets (approximately 79% of the miRNAs), and 147 (approximately 39% of the miRNAs) have at least 10 or more identified interaction sites (approximately 39% of the miRNAs).

When using the Seq${}_{E_{i}}$ datasets as input for Meme, we found a motif associated with each of the miRNAs, with an average width slightly larger than the expected seed length, as indicated in Table 3. On these particular datasets, we therefore always have a perfect score for both precision and recall. This is not surprising since it contains less noise besides the fact that we force the prediction of at least one occurrence per sequence.

We also imposed the same constraint for the human and mouse Seq${}_{W_{i}}$ datasets. In a similar way to the comparison of methods carried out in Section 3.1, we used the CLASH regions to evaluate whether the occurrences of a motif are globally well placed or not. These results are presented in the Supplementary Tables S2 and S3 for each miRNA with at least 2 interaction sites and for each miRNA with at least 10 interaction sites. The results are shown when considering all classes together (first row), and then each one separately. In the Seq${}_{W_{i}}$ datasets, however, the values of the strong version of the F-score decrease rather importantly, and this called for a refinement of the analysis carried out. Additionally, we computed the F-score for each miRNA individually to verify the distribution of these metrics (Supplementary Figure S9).

We checked the F-score computed on the occurrences of a motif (instead of all occurrences of all motifs in the table), which emphasizes the fact that the majority of the motifs, as expected, do not correspond to the interaction sites but to other conserved part of the sequences. There are, however, a few motifs that have near perfect F-scores, which means that they correspond at least partially to interaction sites. These conserved motifs are as expected once again to be found more in the first three classes of the human dataset (because of the seed) and less so in relation to Classes 1 and 2 in the mouse dataset.

We then verified if the best motifs detected by Meme matched the seed region or the miRNA sequence by comparing the matrices of sequences with TomTom [27] from the Meme suite. For this purpose, the seed regions or miRNA sequences were transformed into a matrix with, for each position, a 1 for the nucleotide present at that position and 0 for the others.

**Figure 5:**
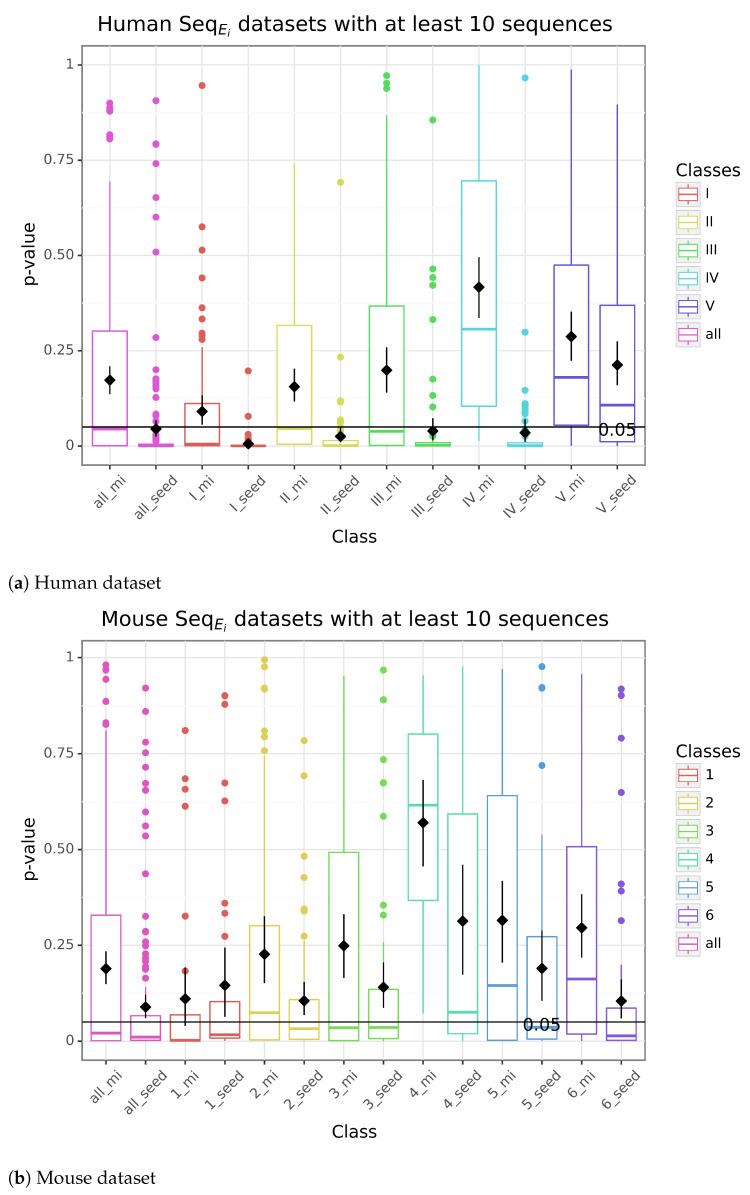
P-values from the comparison of the motifs with the exact interaction sites on the mRNAs as identified on the human and mouse CLASH datasets. The boxplot indicates the distribution of the p-values for each class when comparing the best motif predicted by Meme with either the seed (seed) or the miRNA (mi) on datasets with 10 or more sequences.

Figure 5 shows the boxplot of the p-values given by TomTom when comparing either the seed or the complete miRNA sequence to the motifs predicted by Meme with the site distribution oops (one or plus) when the input contains less noise and only with miRNAs having more than 10 targets.

When using only the region detected by CLASH relative to the miRNAs having more than 10 targets, the classes in which the seed is very well defined (I, II, and III in the human dataset and 1, 2, 3, and 6 in the mouse dataset) showed a better correspondence between the best motif detected and both the seed and the miRNA. Although there is still a correlation between seeds and motifs in Class IV, this trend is lost when using the complete miRNA sequence and not seen at all in both cases for Class V. The same is not observed in the mouse dataset.

Nevertheless, it is interesting to note that by adding more noise to the dataset by either using miRNAs with 2 or more targets or by extending the regions with 200 nucleotides down- and upstream (Supplementary Figures S10C and S11C), the correlation between motifs and seed, or between motifs and miRNA, decreases considerably.

These results could be an indication as to why the seed match might be seen ambiguously as a good or bad feature when predicting targets, especially since we usually do not have the a priori information of the number of targets for a given miRNA and we do not know the correct interaction sites with most sequencing experiments performed for miRNA detection and target prediction.

## 4. Conclusions and Perspectives

In this paper, we revisited the importance of two specific features to predict putative miRNA targets, namely accessibility of the mRNA and seed anchoring, by comparing three state-of-the-art methods (miRanda, Pita, and IntaRNA). To this end, we used datasets based on the CLASH technique, which allowed us to obtain information of both the miRNA along with its mRNA interaction sites.

While the three selected methods rely on seed matches (although calculated differently and not used in the same order among the different features), miRanda is the only method that does not take into account accessibility. IntaRNA was the only method in which we were able to fully exclude both features separately to more fully understand the impact of their absence in the results. As a perspective, it would be very interesting to be able to exclude these two features in other methods in order to investigate whether these and possibly other features are essential to or do not affect the overall results obtained.

Using accessibility to predict miRNA–mRNA interactions is still debated among the research community. However, it is rather safe to say that it does provide evidence for predicting putative targets when used with other traditional features. Overall, by using IntaRNA, we were able to show that the use of this feature in target prediction almost always provided better results in terms of both precision and recall, with few exceptions. Nevertheless, using accessibility as a standalone feature is not advised as it yields too many false positives: there are distinct accessible regions along the mRNA sequences related to other interaction sites with diverse proteins and RNAs.

Unexpectedly, in terms of seed match, when we excluded this feature, we had better or similar results compared to when not using it. In order to understand this ambiguous notion of the importance of the seed match, we verified whether the regions predicted by CLASH as interaction sites of the same miRNA presented a common motif (as predicted by Meme), one that would logically be important for the anchoring of the seed from the miRNA. The idea to use patterns and motifs for the prediction of miRNA targets is not new [28]. Indeed, we found a significant correlation between the seed (or the complete miRNA sequence) and the motifs found in interaction sites from classes in which the seed is well defined (I, II, and III).

Globally, our results showed that increasing the amount of noise around a region that truly interacts with an miRNA negatively impacts the predictions regarding precision and recall for both accessibility and seed match. This information is rather important to keep in mind since the largest portion of the datasets used to predict miRNA targets are rather noisy. In some cases, although we can predict regulatory couples by detecting a correlation of expression between a given miRNA and its putative mRNA targets, most sequencing experiments do not provide the exact site of interaction between a miRNA and its mRNA target.

Moreover, despite the results obtained in this first analysis on the possible usefulness of exploiting intra-species conservation and the associated over-representation of motifs to infer new interaction sites and thus also new miRNAs, it might be interesting to continue investigating this idea in the future, perhaps with a refinement on how such motifs are inferred. One path that might be worth exploring would be limiting such motif inference to mRNA regions with high accessibility, thereby potentially reducing the amount of noise in the input given to tools such as Meme.

## Figures and Tables

**Table 1:** Number of interactions for each human dataset, in total and by region and class.

Number of interactions for the Seq${}_{C}$ dataset									
**Total**	**5** ${}^{\prime }$ **UTR**	**CDS**	**3** ${}^{\prime }$ **UTR**	**Other mRNA regions**	**Class I**	**Class II**	**Class III**	**Class IV**	**Class V**
18,497	868	11,112	6093	424	3590	3290	4629	3382	3606
Number of interactions for the Seq${}_{W}$ dataset									
**Total**	**5${}^{\prime }$UTR**	**CDS**	**3${}^{\prime }$UTR**	**Other mRNA regions**	**Class I**	**Class II**	**Class III**	**Class IV**	**Class V**
15,754	291	10,304	4916	243	3095	2833	3970	2890	2966

**Table 2:** Number of interactions for each mouse dataset, in total and distributed by region and class.

Number of interactions for the Seq${}_{C}$ dataset										
**Total**	**3** ${}^{\prime }$ **UTR**	**CDS**	**5** ${}^{\prime }$ **UTR**	**Other mRNA regions**	**Class 1**	**Class 2**	**Class 3**	**Class 4**	**Class 5**	**Class 6**
13,071	4843	7136	187	905	1631	2672	2269	1117	2363	3019
Number of interactions for the Seq${}_{W}$ dataset										
**Total**	**3** ${}^{\prime }$ **UTR**	**CDS**	**5** ${}^{\prime }$ **UTR**	**Other mRNA regions**	**Class 1**	**Class 2**	**Class 3**	**Class 4**	**Class 5**	**Class 6**
11,581	3817	6917	102	745	1428	2366	1987	976	2129	2695

**Table 3:** Average width (column “Av. width”) of the best motif of width between 6 and 23 found by Meme using the oops site distribution on the sequences given by CLASH. The column “#Seqs” corresponds to the total number of sequences for each subset.

Human Dataset				
	Seq${}_{E_{i}}$		Seq${}_{W_{i}}$	
**Classes**	**Av. width**	**#Seqs**	**Av. width**	**#Seqs**
all	11.31	18,431	14.57	15,670
I	10.36	3531	13.77	3030
II	11.50	3224	14.83	2770
III	11.84	4560	15.11	3907
IV	11.0	3318	14.29	2828
V	10.15	3534	14.25	2898
Mouse Dataset				
	Seq${}_{E_{i}}$		Seq${}_{W_{i}}$	
**Classes**	**Av. width**	**#Seqs**	**Av. width**	**#Seqs**
all	10.0	12,992	14.72	11,500
1	9.45	1556	13.18	1357
2	9.77	2588	13.40	2287
3	9.54	2182	14.01	1908
4	8.92	1061	14.35	922
5	9.90	2298	13.62	2057
6	9.42	2947	14.09	2624

## Data Availability

The data may be recovered following the explanations we gave in the Materials and Methods section. Should someone want the data directly, the authors can provide such data upon request.

## Supplementary Materials

The following supporting information can be downloaded at: https://www.mdpi.com/article/10.3390/genes14030664/s1, Figure S1: Performance of the three methods considering accessibility and seed match on the mouse Seq${}_{W}$ and Seq${}_{C}$ datasets. Figure S2: Performance of IntaRNA considering or not accessibility and seed match on the mouse Seq${}_{W}$ and Seq${}_{C}$ datasets. Figure S3: Performance of the three methods considering accessibility and seed match on the human Seq${}_{W}$ and Seq${}_{C}$ datasets. Figure S4: Performance of IntaRNA considering or not accessibility and seed match on the human Seq${}_{W}$ and Seq${}_{C}$ datasets. Figure S5: Normalised accessibility of all classes on Seq${}_{W}$ of the human dataset. Figure S6: Normalised Accessibility of all classes on Seq${}_{W}$ of the mouse dataset. Figure S7: One randomly selected interaction for each class on each location on the mouse dataset. Figure S8: One randomly selected interaction for each class on each location on the mouse dataset. Figure S9: Histogram representing the number of miRNAs by f-score on Seq${}_{W_{i}}$ for each dataset and for each class. Figure S10: P-value from the comparison of the motifs with human Seq${}_{E_{i}}$ and Seq${}_{W_{i}}$ datasets. Figure S11: P-value from the comparison of the motifs with mouse Seq${}_{W_{i}}$ and Seq${}_{W_{i}}$ datasets. Table S1: Version and execution details for each tool. Table S2: Best motif of width between 6 and 23 found by Meme using the oops site distribution. Table S3: Best motif of width between 6 and 23 found by Meme using the oops site distribution.
