# Supplementary material for: MicroRNA Target Identification: Revisiting Accessibility and Seed Anchoring
Source: Genes (Basel). 2023 Mar 7;14(3):664. doi: 10.3390/genes14030664 (PMC10048102; doi:10.3390/genes14030664)
Supplement: Supplementary file 1 [file genes-14-00664-s001.zip › genes-2094043-supplementary.pdf]

Supplementary tables and figures

Table S1. Version and execution details for each tool.

Methods	Version	Execution
MIRANDA	MIRANDA v3.3a	MIRANDA mir_file mrna_file
INTARNA	INTARNA 2.3.1 using ViennaPackage 2.4.11 and boost 1.68.0	INTARNA -q mir_file -t utr_file –out STDOUT –outMode C -n nb_predictions
PITA	Version 2008 64bit	PITA -utr utr_file -mir mir_file -prefix output
MEME	MEME suite 5.4.1	MEME sequence_file -dna -oc script_path -nostatus -time 14400 -mod oops -nmotifs nb_motifs -minw min_size_motif -maxw max_size_motif -objfun classic -revcomp -markov_order 0 -text
Vienna Package	2.4.15	

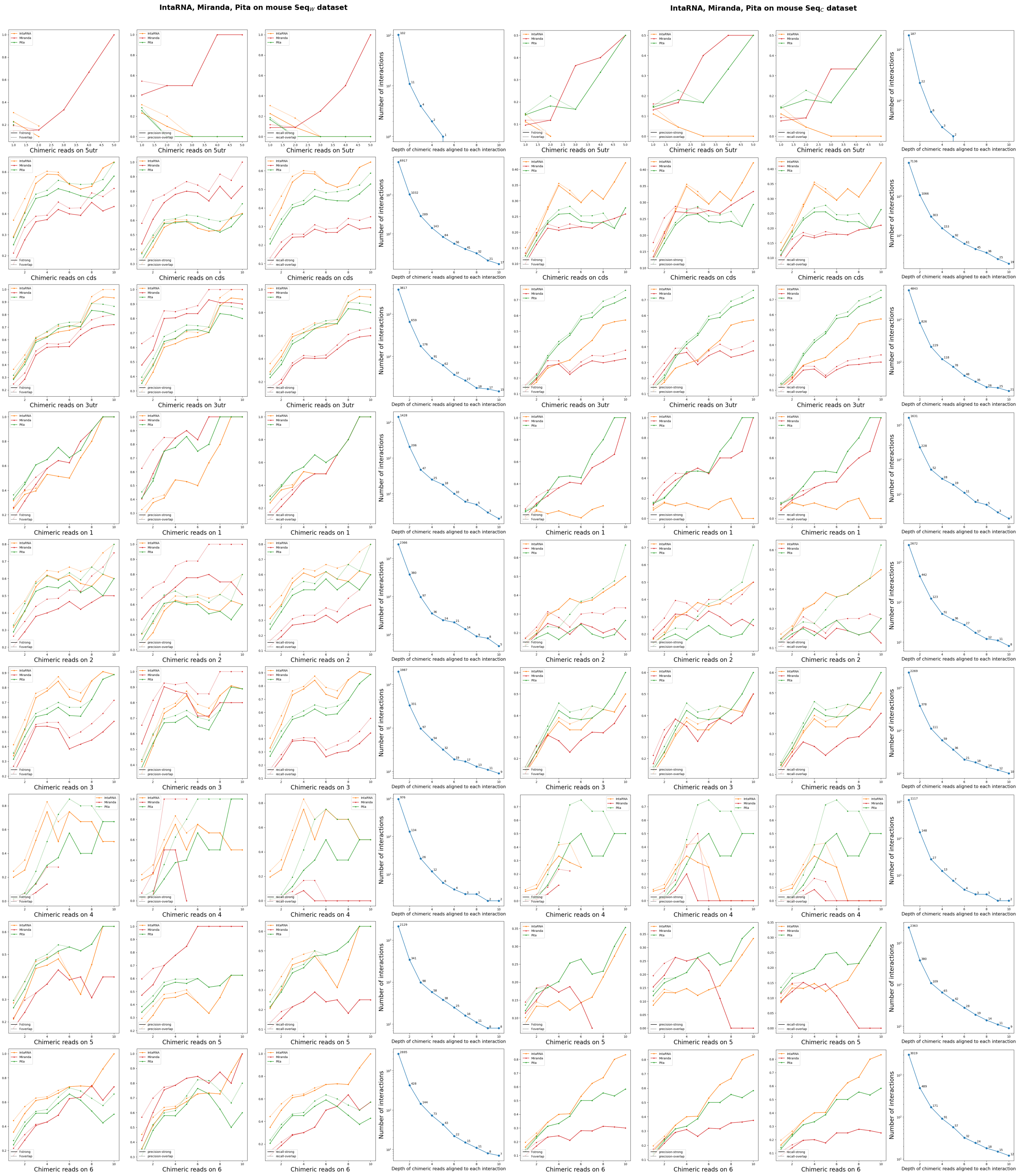

Figure S1. Performance of the three methods considering accessibility and seed match on the mouse Seq$_W$ and Seq$_C$ datasets.

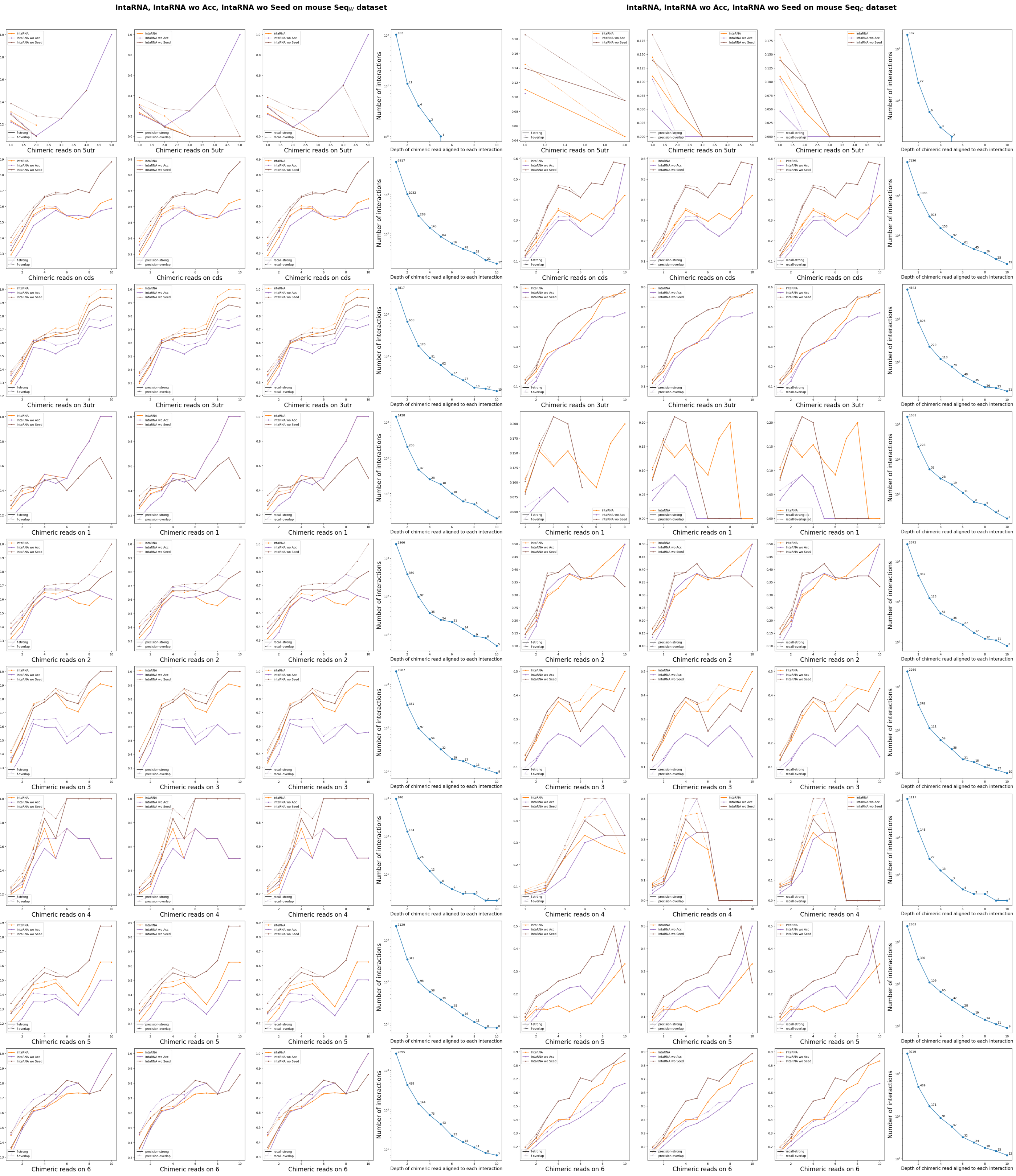

Figure S2. Performance of IntaRNA considering or not accessibility and seed match on the mouse Seq$_W$ and Seq$_C$ datasets. The term wo in the Figure stands for WithOut.

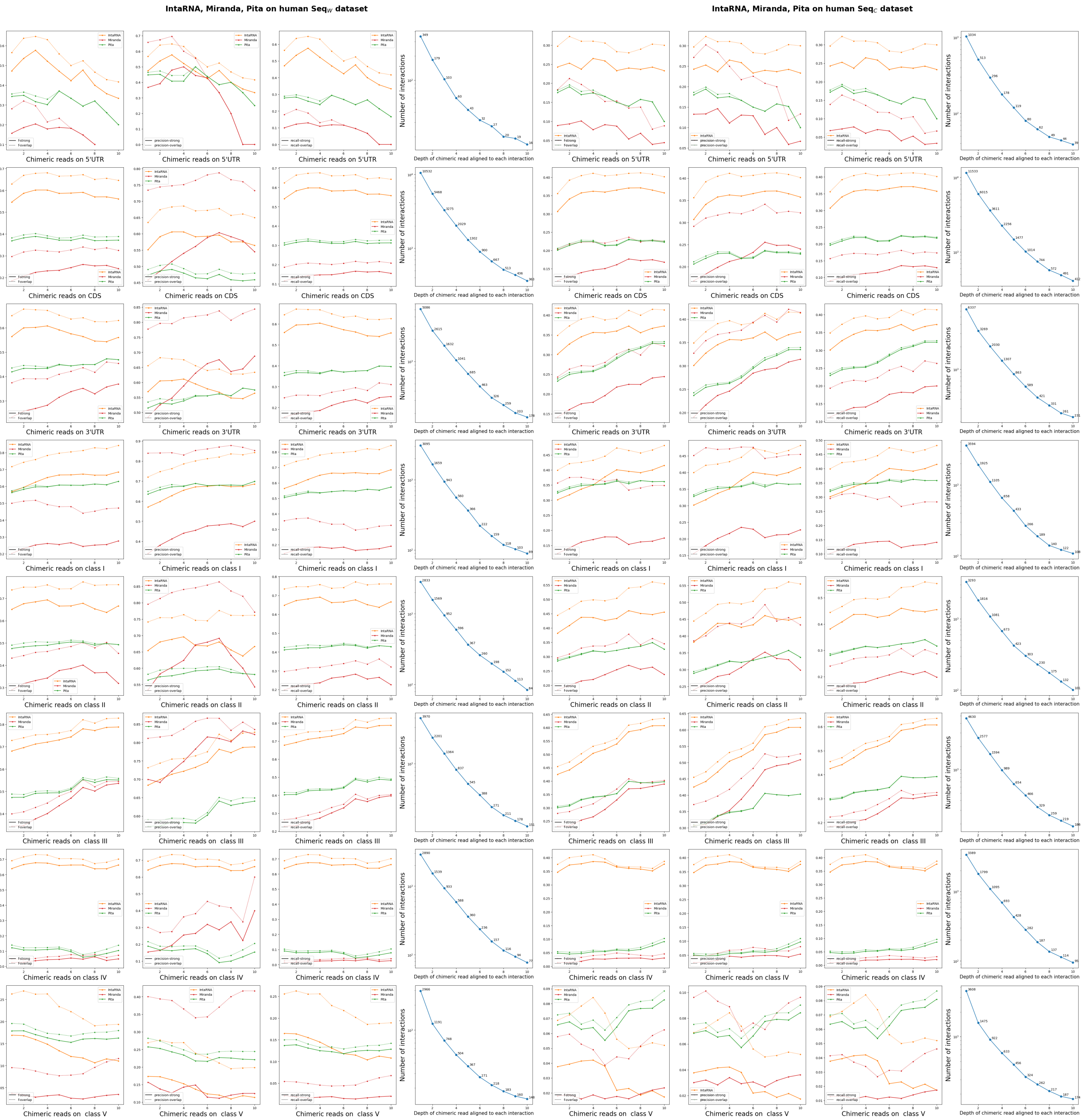

Figure S3. Performance of the three methods considering accessibility and seed match on the human Seq$_W$ and Seq$_C$ datasets.

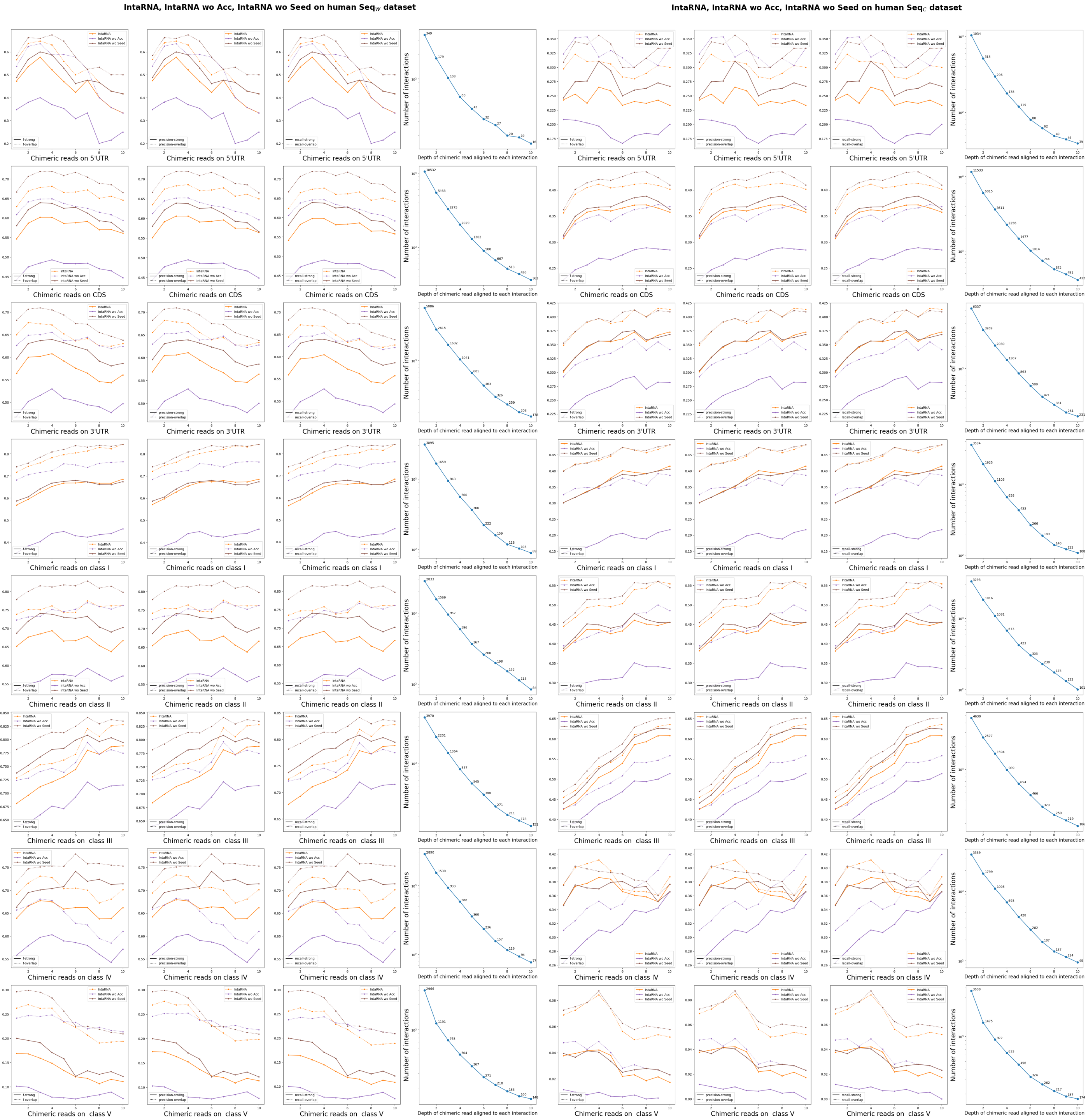

Figure S4. Performance of INTARNA considering or not accessibility and seed match on the human Seq$_W$ and Seq$_C$ datasets. The term wo in the Figure stands for WithOut.

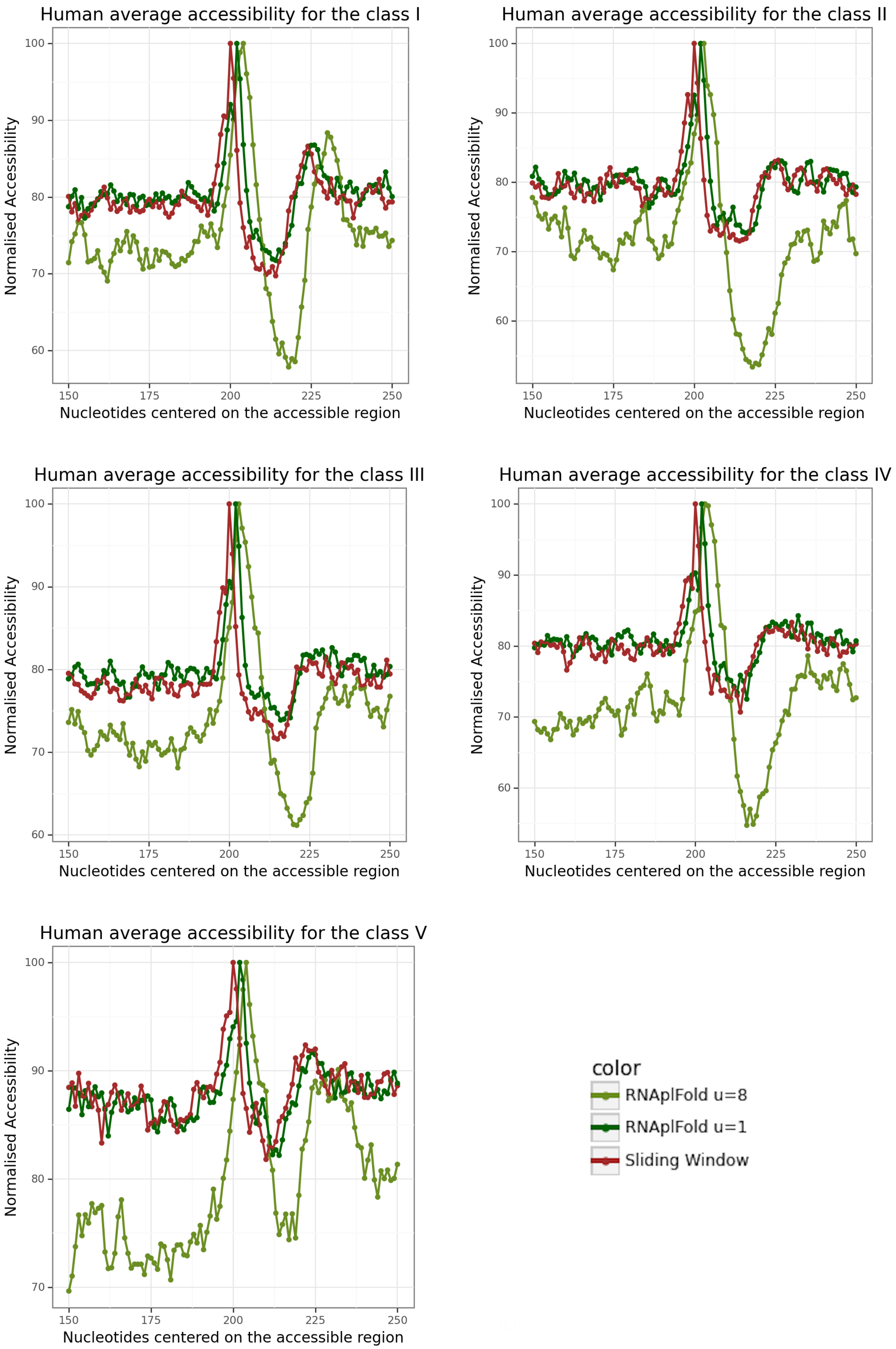

Figure S5. Normalised accessibility of all classes on Seq_W of the human dataset. In red, the normalised accessibility when considering only the optimal structure with a sliding window. In darkgreen and in olivegreen, the normalised accessibility computed with RNAPLFOLD with an accessible window u of 1 and 8.

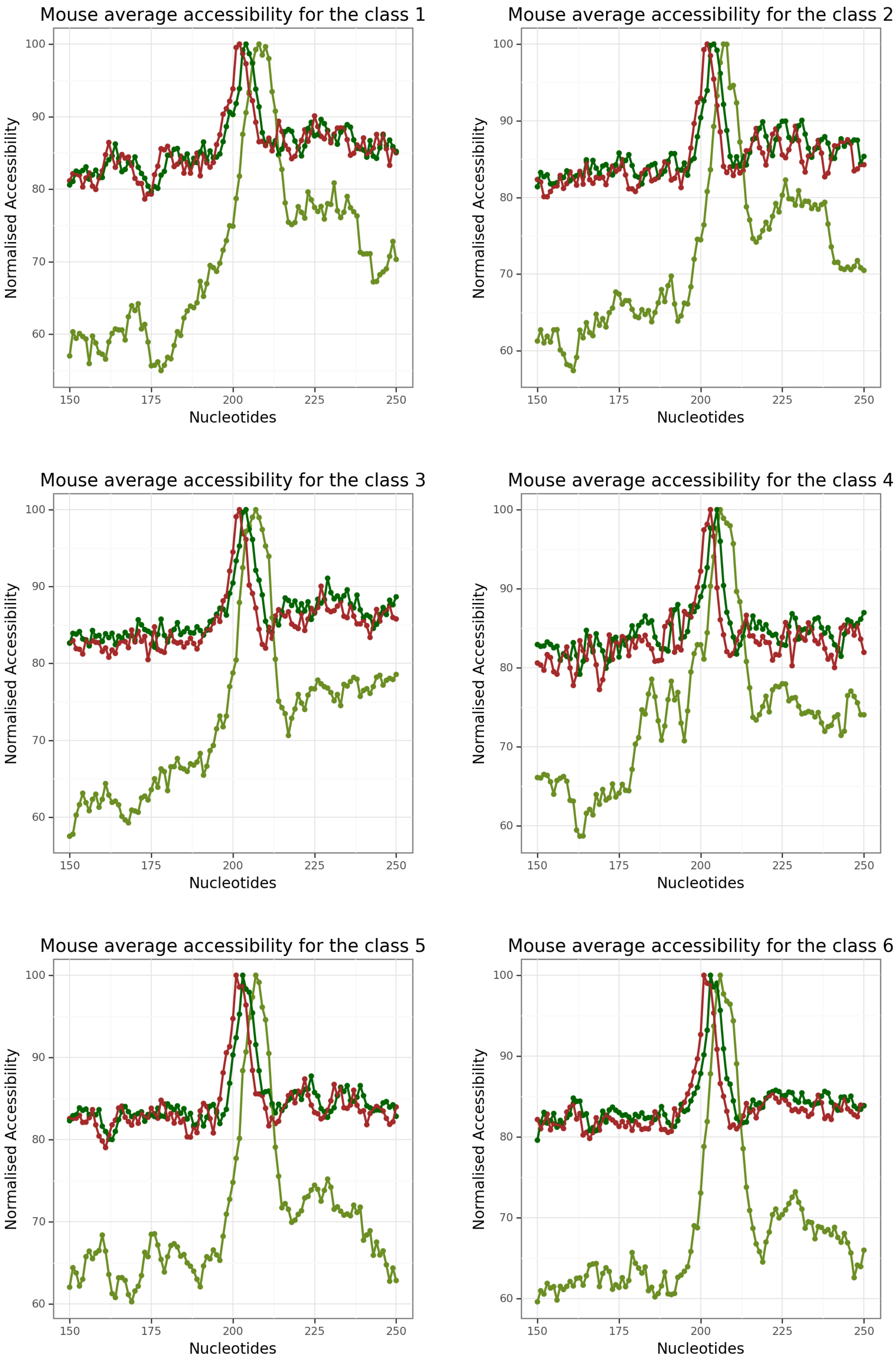

Figure S6. Normalised Accessibility of all classes on Seq_W of the mouse dataset. In red, the normalised accessibility when considering only the optimal structure with a sliding window. In darkgreen and in olivegreen, the normalised accessibility computed with RNAPLFOLD with an accessible window u of 1 and 8.

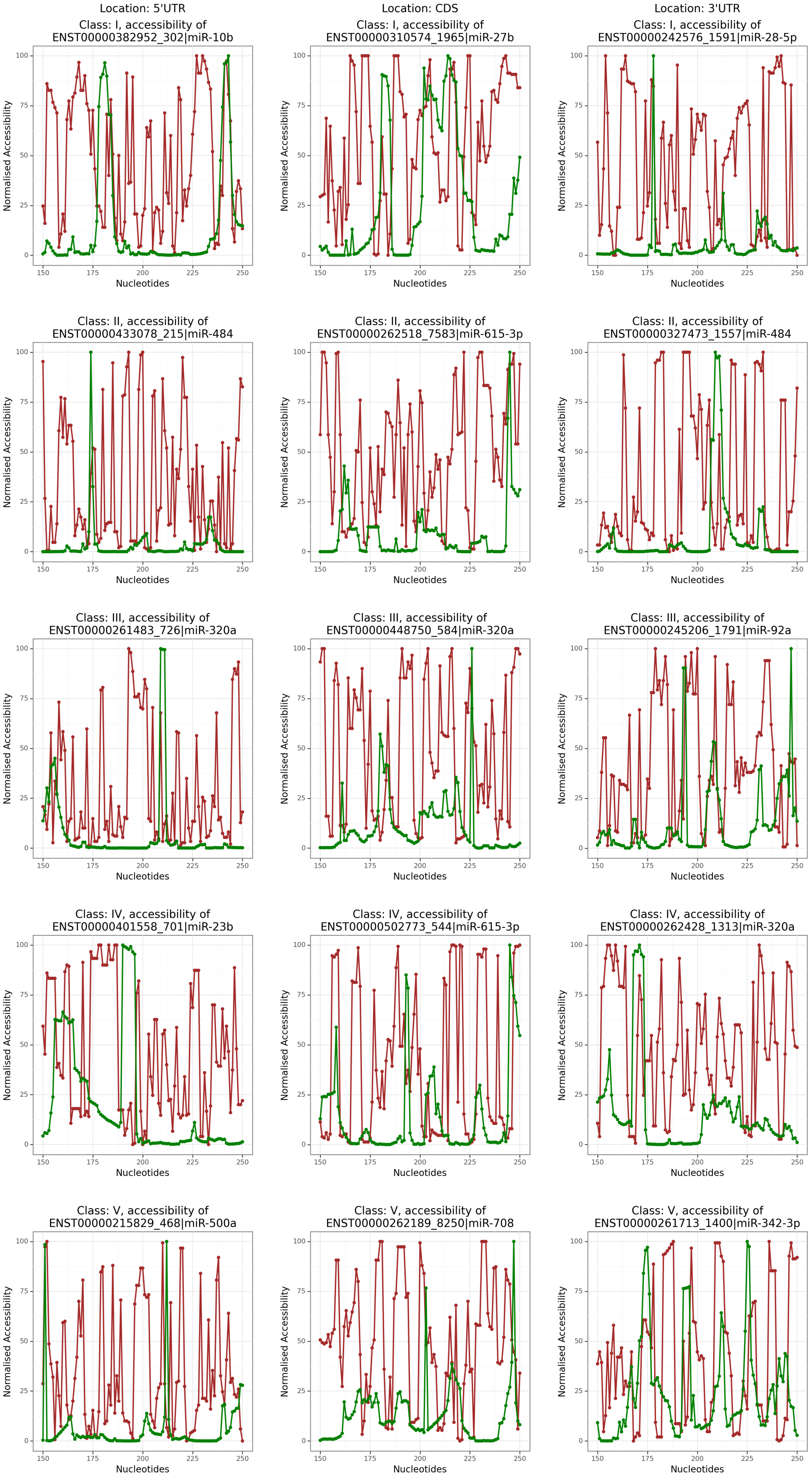

Figure S7. One randomly selected interaction for each class on each location on the human dataset. In red, the normalised accessibility when considering only the optimal structure with a sliding window. In green, the accessibility computed with RNAPLFOLD with an accessible window u of 8.

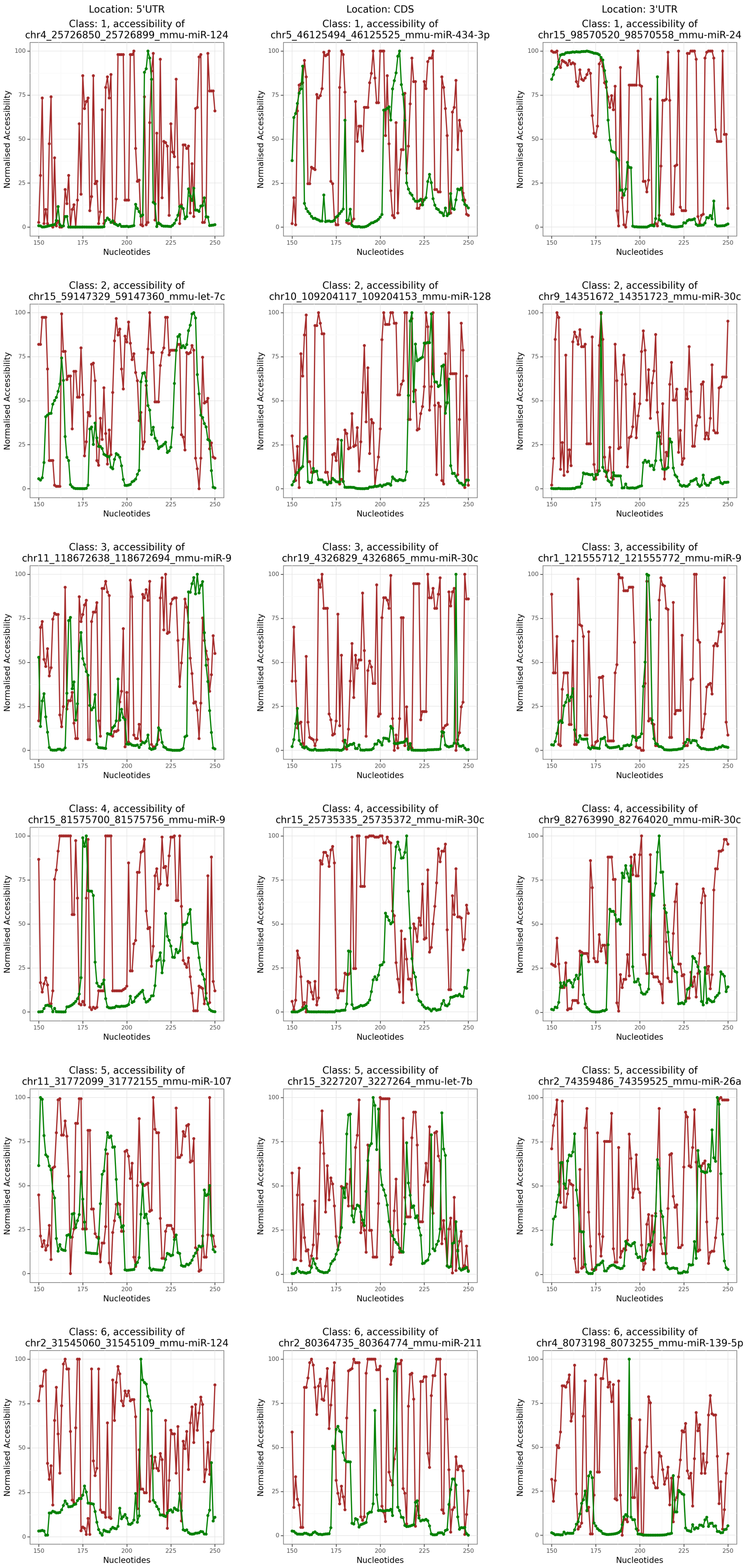

Figure S8. One randomly selected interaction for each class on each location on the mouse dataset. In red, the normalised accessibility when considering only the optimal structure with a sliding window. In green, the accessibility computed with RNAPLFOLD with an accessible window u of 8.

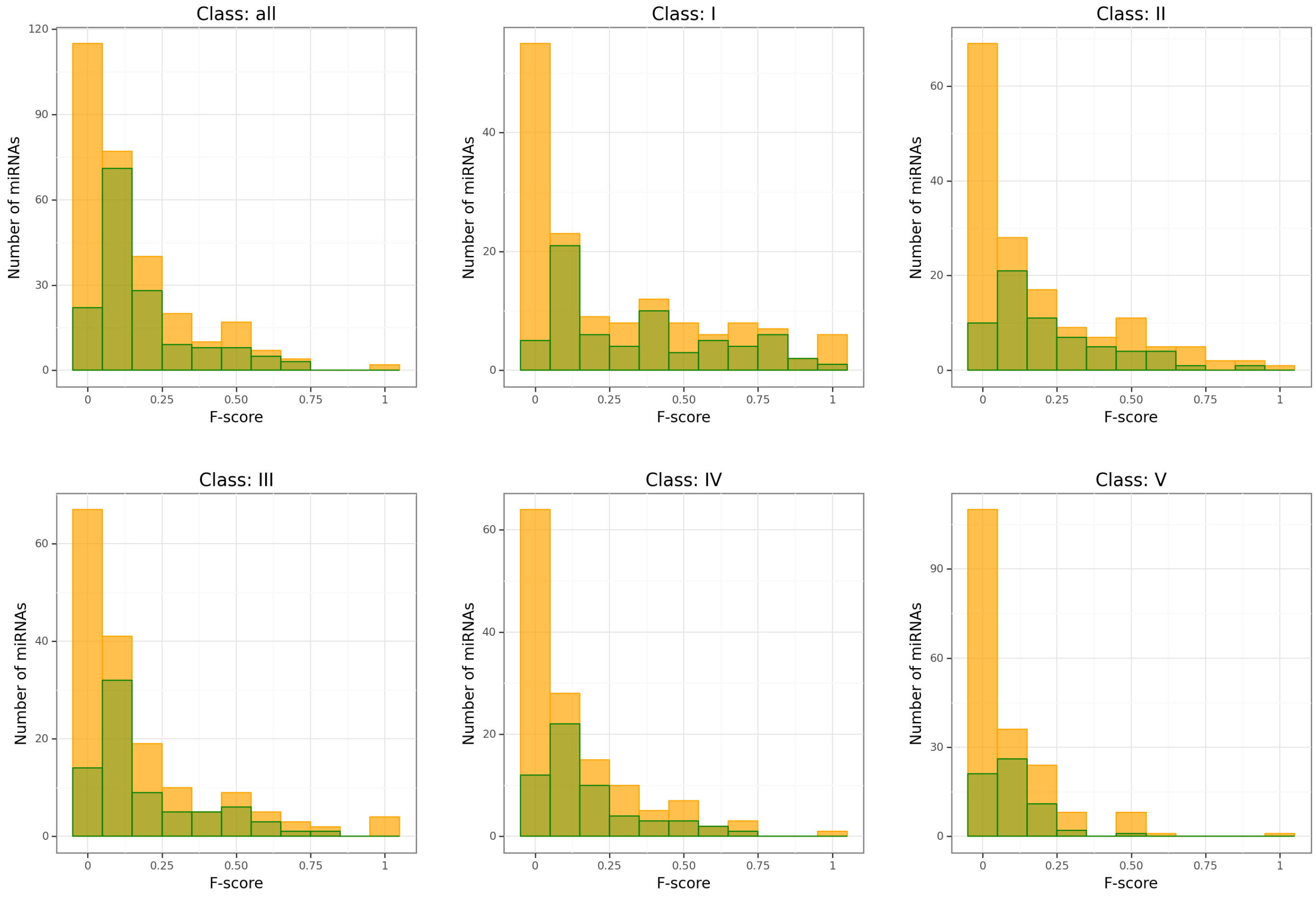

(a) Human dataset

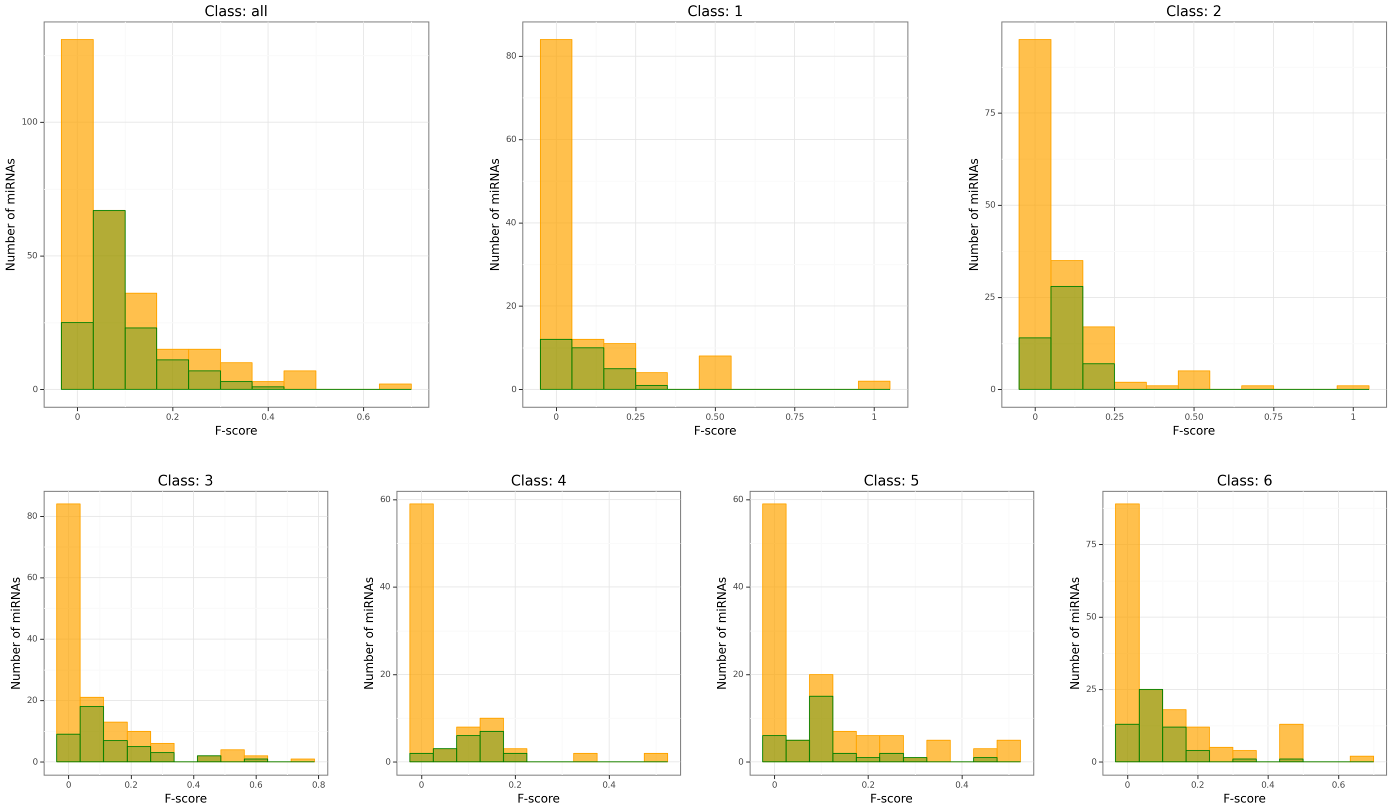

(b) Mouse dataset

Figure S9. Histogram representing the number of miRNAs by f-score on Seq_{W_i} for each dataset and for each class. In orange, are the datasets with at least 2 targets, and in green those with at least 10 targets. Note that the green dataset is a subset of the orange one which implies that the green number of miRNAs is equal or smaller than in the orange.

Table S2. Best motif of width between 6 and 23 found by MEME using the oops site distribution. The column "Av. width" corresponds to the average length of the best motif found while the column "#Seqs" corresponds to the total number of sequences for each subset (Total, and then Class I to Class V).

Input: Seq_{W_i} on human and associated with miRNAs targeting at least 2 mRNAs									
	Global numbers			Occurrences validation				F-score	
**Class**	**#miRNAs**	**Av. width**	**#Seqs**	**TP$_{strong}$**	**FP**	**Over-start**	**Over-end**	**F-strong**	**F-overlap**
Total	292	14.58	15670	2421	13249	607	531	0.15	0.23
I	144	13.77	3030	822	2208	100	97	0.27	0.34
II	156	14.84	2770	531	2239	113	86	0.19	0.26
III	165	15.12	3907	729	3178	161	119	0.19	0.26
IV	135	14.30	2828	447	2381	124	93	0.16	0.23
V	188	14.26	2898	257	2641	112	128	0.09	0.17
Input: Seq_{W_i} on human and associated with miRNAs targeting at least 10 mRNAs									
	Global numbers			Occurrences validation				F-score	
**Class**	**#miRNAs**	**Av. width**	**#Seqs**	**TP$_{strong}$**	**FP**	**Over-start**	**Over-end**	**F-strong**	**F-overlap**
Total	154	16.01	15144	2362	12782	595	517	0.16	0.23
I	67	14.97	2729	755	1974	91	85	0.28	0.34
II	64	16.88	2382	464	1918	101	72	0.19	0.27
III	76	16.61	3543	672	2871	145	112	0.19	0.26
IV	57	16.00	2520	412	2108	118	88	0.16	0.25
V	61	15.93	2372	205	2167	99	108	0.09	0.17

Table S3. Best motif of width between 6 and 23 found by MEME using the oops site distribution. The column "Av. width" corresponds to the average length of the best motif found while the column "#Seqs" corresponds to the total number of sequences for each subset (Total, and then Class 1 to Class 6).

Input: Seq_{W_i} on mouse and associated with miRNAs targeting at least 2 mRNAs									
	Global numbers			Occurrences validation				F-score	
**Class**	**#miRNAs**	**Av. width**	**#Seqs**	**TP**$_{strong}$	**FP**	**Over-start**	**Over-end**	**F-strong**	**F-overlap**
All	285	14.72	9641	776	8865	302	320	0.08	0.15
1	121	13.18	1357	112	1245	34	32	0.08	0.13
2	157	13.40	2287	213	2074	57	61	0.09	0.14
3	143	14.01	1908	208	1700	55	55	0.11	0.17
4	87	14.36	922	81	841	30	33	0.09	0.16
5	117	13.62	2057	202	1855	41	63	0.10	0.15
6	168	14.10	2624	212	2412	88	101	0.08	0.15
Input: Seq_{W_i} on mouse and associated with miRNAs targeting at least 10 mRNAs									
	Global numbers			Occurrences validation				F-score	
**Class**	**#miRNAs**	**Av. width**	**#Seqs**	**TP**$_{strong}$	**FP**	**Over-start**	**Over-end**	**F-strong**	**F-overlap**
All	136	15.37	8982	719	8263	282	298	0.08	0.14
1	28	15.36	1034	86	948	26	28	0.08	0.14
2	49	14.98	1882	179	1703	49	55	0.10	0.15
3	45	15.36	1495	179	1316	42	42	0.12	0.18
4	20	15.65	678	71	607	21	28	0.10	0.18
5	33	15.06	1684	159	1525	33	57	0.09	0.15
6	56	16.55	2193	171	2022	74	89	0.08	0.15

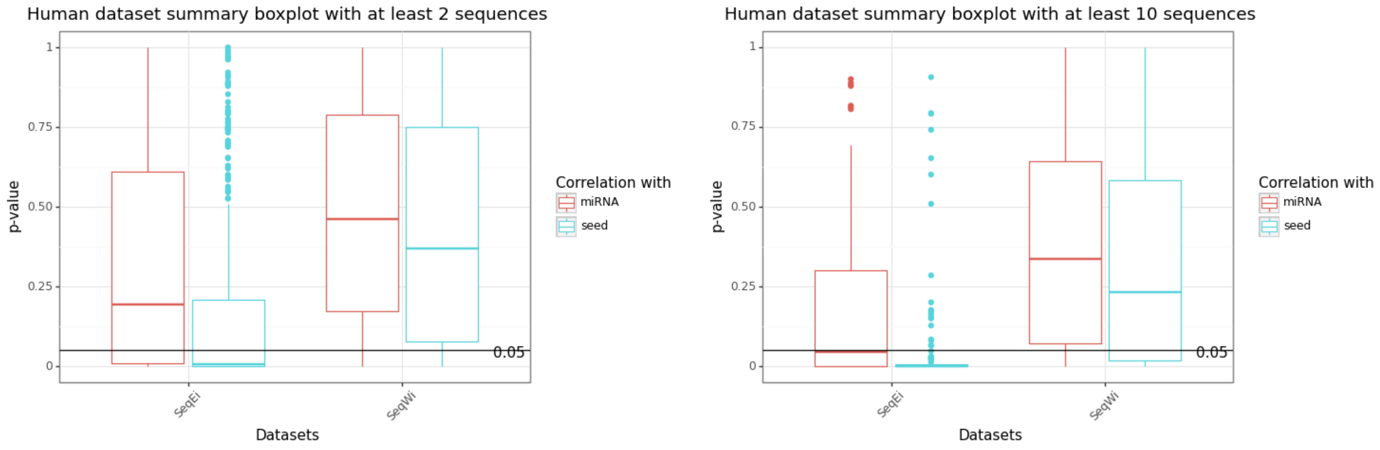

(a) Correlation with the miRNA and the seed summarized.

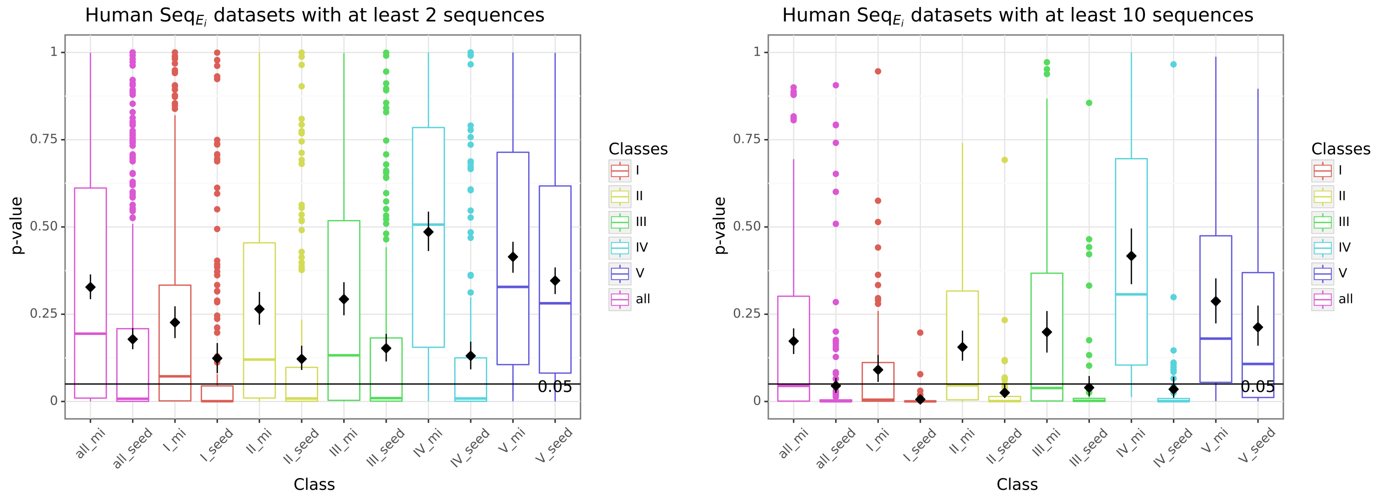

(b) P-value from the comparison of the motifs with the sequences given by CLASH. The boxplot indicates the distribution of the p-values for each class when comparing the best motif predicted by MEME with either the seed (seed) or the miRNA (mi) on datasets with more than 2 or 10 sequences.

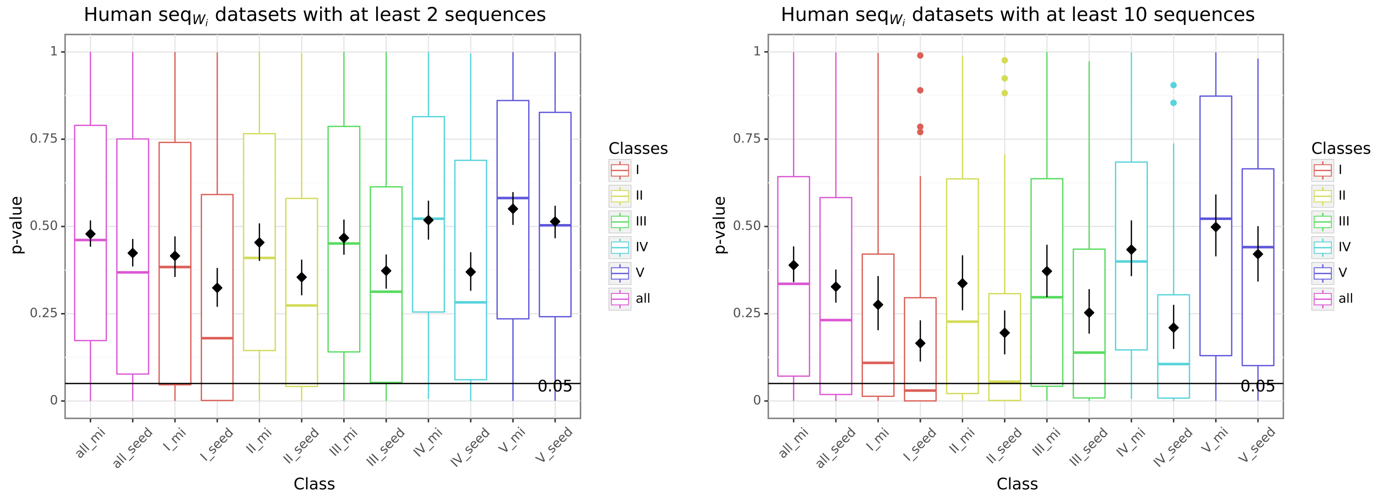

(c) P-value from the comparison of the motifs with Seq_{W_i} datasets. The boxplot indicates the distribution of the p-values for each class when comparing the best motif predicted by MEME with either the seed (seed) or the miRNA (mi) on datasets with more than 2 or 10 sequences.

Figure S10. Boxplots on the human dataset.

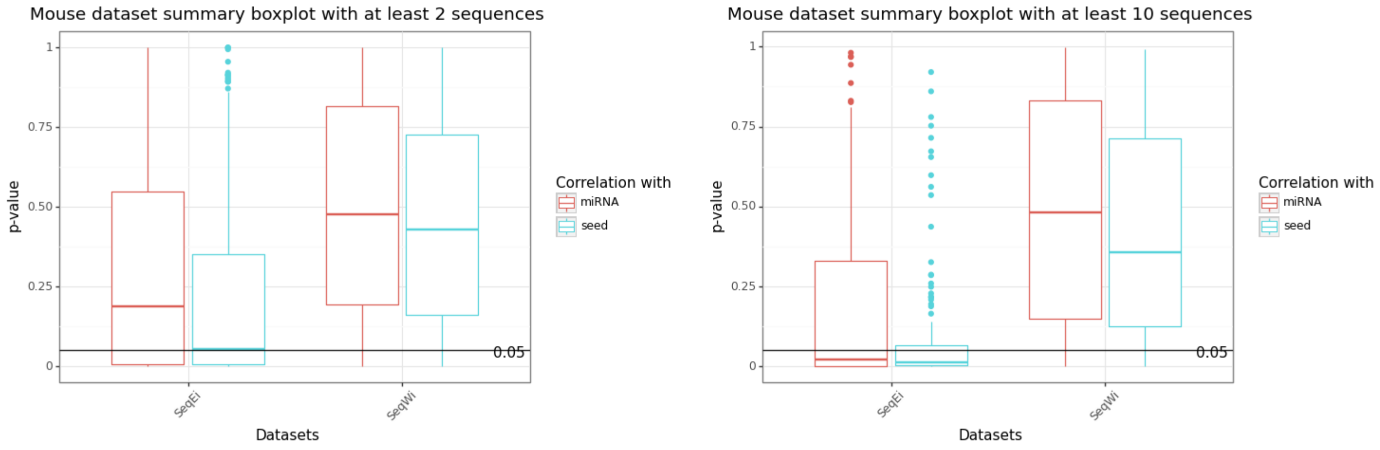

(a) Correlation with the miRNA and the seed summarized.

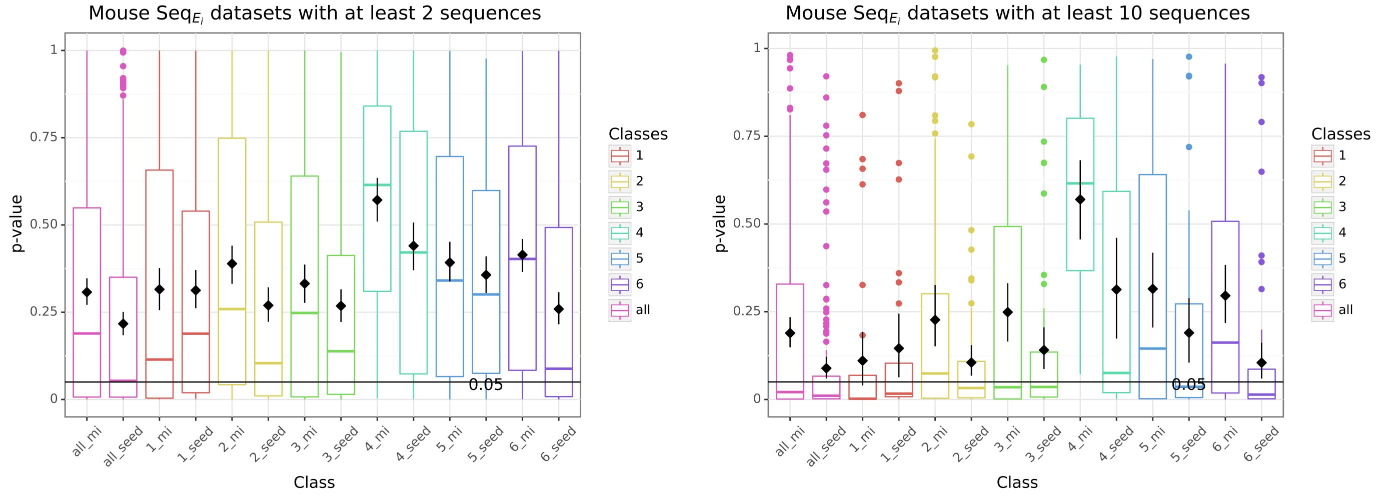

(b) P-value from the comparison of the motifs with the sequences given by CLASH. The boxplot indicates the distribution of the p-values for each class when comparing the best motif predicted by MEME with either the seed (seed) or the miRNA (mi) on datasets with more than 2 or 10 sequences.

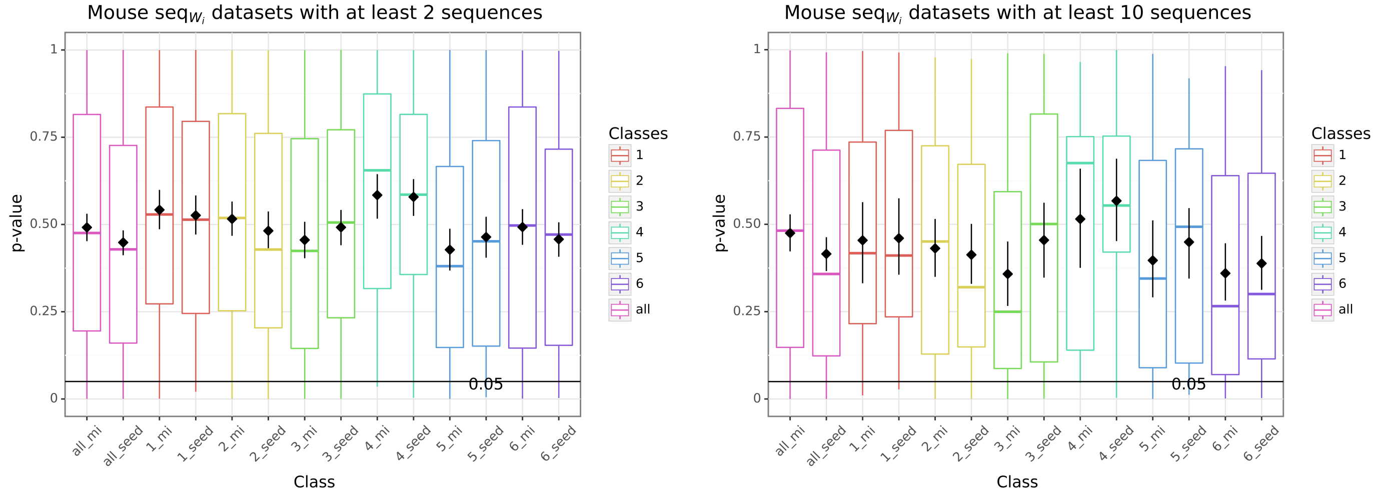

(c) P-value from the comparison of the motifs with Seq_{W_i} datasets. The boxplot indicates the distribution of the p-values for each class when comparing the best motif predicted by MEME with either the seed (seed) or the miRNA (mi) on datasets with more than 2 or 10 sequences.

Figure S11. Boxplots on the mouse dataset.
